# Double-Layer Metasurface Integrated with Micro-LED for Naked-Eye 3D Display

**DOI:** 10.3390/nano14201624

**Published:** 2024-10-10

**Authors:** Qinyue Sun, Zhenhuan Tian, Chuangcheng Xu, Angsu Yu, Feng Li, Feng Yun

**Affiliations:** 1 Shaanxi Provincial Key Laboratory of Photonics & Information Technology, Xi’an Jiaotong University, Xi’an 710049, China; sunqy@stu.xjtu.edu.cn (Q.S.); xcc1025@stu.xjtu.edu.cn (C.X.); 2201422102@stu.xjtu.edu.cn (A.Y.); felix831204@xjtu.edu.cn (F.L.); 2 Solid-State Lighting Engineering Research Center, Xi’an Jiaotong University, Xi’an 710049, China

**Keywords:** naked-eye 3D display, micro-LED, metasurface, large field of view, high-resolution

## Abstract

Naked-eye 3D micro-LED display combines the characteristics of 3D display with the advantages of micro-LED. However, the 3D micro-LED display is still at the conceptual stage, limited by its intrinsic emission properties of large divergence angle and non-coherence, as well as difficulties in achieving large viewing angles with high luminous efficiency. In this work, we propose a double-layer metasurface film integrating functions of collimation with multiple deflections, constituting a micro-LED naked-eye 3D display system. The system is characterized through numerical simulations using the 3D finite-difference time-domain method. The simulation results show that the double-layer metasurface film restricts 90% of the emitted light of the micro-LED to the vicinity of the 0° angle, improving its spatial coherence. Subsequently, a large-angle, low-crosstalk outgoing from −45° to 45° is achieved, while providing a deflection efficiency of over 80% and a pixel density of up to 605. We believe this design provides a feasible approach for realizing naked-eye 3D micro-LED displays with a large field of view, low crosstalk, and high resolution.

## 1. Introduction

Three-dimensional (3D) displays can provide image content closer to the real world, and thus have received extensive attention from researchers in recent years. The naked-eye 3D display does not require additional equipment, so it has become an inevitable trend of 3D display [1]. At present, the auto-stereoscopic display is a mainstream naked-eye 3D display because of its compatibility with the existing flat panel display technology supported by a matured production chain [2]. It combines the viewing angle control board with the flat panel display screen to discretize the continuous light field of a 3D object into multiple viewing angle light fields, so that the multi-view image with continuous motion parallax and binocular parallax effects can approximate the expression of 3D object information [3].

In auto-stereoscopic 3D display technology, existing implementations include parallax barriers [4,5], lens arrays [6,7,8], and directional backlighting techniques [9,10], etc. For example, Peter Krebs et al. designed a directional backlight display system with high resolution and high brightness uniformity using left- and right-distributed strip light sources, lens arrays, and LCD panels [11]. However, most of the 3D display technologies based on these solutions have restricted viewing positions, low resolution, and serious crosstalk, which greatly reduces the effectiveness of 3D displays [3]. Therefore, how to balance spatial resolution, angular resolution, field of view, and light utilization efficiency remains to be explored. In addition, these technologies are all based on LCD displays, and the inherent defects of LCD itself, such as limited viewing angle, insufficient brightness, limited color performance, and high energy consumption, have also constrained the development of 3D displays.

With the rapid development of display technology, GaN-based micro-LED is considered the most promising display technology, with advantages such as high contrast, low power consumption, and high response frequency [12,13,14]. Micro-LED devices with controllable light distribution and direction provide a new solution for well-designed viewing angle modulation boards. However, micro-LED emits light in a Lambertian distribution with a wide divergence angle higher than ±60°, resulting in optical crosstalk between adjacent pixels, which affects the clarity of the display [15,16]. Improving the collimation of micro-LED light emission can reduce the optical crosstalk between adjacent pixels. Currently, methods to improve the collimation of micro-LEDs include resonant cavities (RC) [2,17,18], lens collimation [19,20], and light-blocking patterns between pixels [21]. Lens collimation based on geometrical optics can improve the coupling efficiency to some extent [20]. However, the designed lenses tend to be much larger than the size of the micro-LED, which is not conducive to miniaturization and integration. The use of distributed Bragg reflectors and bottom mirrors to form a resonant cavity well limits the angular distribution of micro-LEDs [22]. However, it is highly sensitive to the cavity length and emission wavelength, and has a complex structure with low collimation efficiency. Therefore, there is still an urgent need for a collimation scheme that can provide excellent collimation performance and a miniaturized size for micro-LED display.

Metasurfaces [23,24,25,26] are ultrathin artificial structures at a subwavelength scale that can manipulate the amplitude, phase, or polarization of light waves by adjusting the parameters of the unit structure, enabling beam steering [24,27], holography [28], polarization conversion [29,30], and so on. Compared with geometric optical elements and diffractive optical elements, metasurfaces have the advantages of high design freedom and subwavelength control accuracy. Integrating metasurfaces into micro-LED structures can maximize their compatibility with semiconductor processes [15]. However, the concept of a collimated metasurface integrated with micro-LEDs has only been proposed conceptually, and has not yet been thoroughly explored.

Additionally, the application of micro-LEDs in 3D display is also limited by the difficulty in achieving large viewing angles, in which multiple viewing angles need to be obtained. Even though the metasurface has been used to perform directional deflection of parallel light sources at specific angles (10°, 20°, 30°) [1,17,18], a single deflection angle can only support one viewing point, resulting in image artifacts and crosstalk for viewers, greatly reducing the overall viewing experience [8]. By scarifying the resolution, the viewing angle can be slightly expanded, but not enough. Therefore, it is necessary to explore control methods that can accurately achieve the deflection angles, to achieve large fields of view and high-efficiency emission of micro-LEDs.

In this paper, we propose an optical system for naked-eye 3D display, which integrates micro-LEDs with a double-layer metasurface. This metasurface can achieve a dual function in collimation and multiple deflections. Firstly, we design a single-layer metasurface for multiple deflections. By dividing the subpixels into multiple modules, it achieves a large viewing angle from $-50^{{}^{\circ}}\sim 50^{{}^{\circ}}$ with 8 points of view directed out. The pixel density of up to 300 PPI and efficiency of >80% demonstrate its excellent performance in balancing a large field of view, high resolution, and high emission efficiency. Then, a double-layer metasurface structure is introduced for micro-LED light sources with a Lambertian distribution pattern. The collimated metalens setting under the deflection metasurface can directly convert the outgoing light from a point source to parallel light. This double-metasurface is independent of the light source without a resonant cavity, thus it simplifies the manufacturing process and allows integration with existing displays. This optical system can achieve a large angle from $-45^{{}^{\circ}}\sim 45^{{}^{\circ}}$, an efficiency greater than 80%, and a pixel density of up to 605 PPI. This solves the problem of large crosstalk caused by beam interference between pixels when conventional micro-LEDs are used as display pixels due to their large divergence angle. The large viewing angle and the high luminous efficiency verify the feasibility of the metasurface in application of naked-eye 3D micro-LED display.

## 2. Structures and Methods

### 2.1. The Principle of Naked-Eye 3D Display

**Figure 1:**
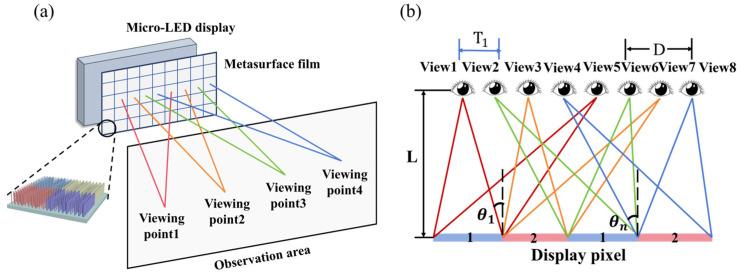
(**a**) Schematic diagram of the micro-LED naked-eye 3D display system based on metasurface. (**b**) Schematic of multi-view subpixel imaging.

The design of 3D displays is based on the principle of binocular vision in the human eye. For a good implementation of a naked-eye 3D display, the parallax image of the combined display must be able to be transmitted correctly to each observer’s eye. Figure 1a demonstrates the micro-LED naked-eye 3D display system based on metasurfaces. Covering the designed metasurface film on the display panel modulates the outgoing light to transmit parallax images from different positions in different directions. In turn, the parity pixels on the display enter the viewer’s left and right eyes, forming a stereoscopic display after being processed by the brain’s visual system. The light passing through the metasurface needs to be focused at different positions according to the viewing position, and the spacing of the light-emitting peaks of the nanostructured subpixel units needs to meet the spacing requirements of the human eye. This can be achieved by controlling the deflection angle of the emitted light from subpixel A, subpixel B, and so on.

Figure 1b shows the schematic structure when the number of subpixels is 4 and the number of viewpoints is 8. By designing the metasurface, it is ensured that the array of subpixels labeled with the number 1 delivers the image to the left eye, and the array of subpixels labeled with the number 2 delivers the image to the right eye. $\theta_{1}$, $\theta_{n}$ denotes the direction angles of the maximum intensity of the micro-LED pixels, and $T_{n}$ denotes the horizontal distance between the center position of the light-emitting point and the viewpoint. From the geometric relation, we have $\theta_{1}=arctan(T_{1}/L)$, and the same reasoning can be extended to obtain

(1) $$\theta_{n}=arctan{(T}_{n}/L)(n=0,1,2,\ldots )$$

where L is the distance between the observer and the display. D represents the distance between the observer’s eyes. In the actual imaging model, the image is most clearly viewed when the viewing point is located on the center depth plane (CDP). The distance from CDP to display is the optimal viewing distance. Therefore, by adjusting the light-emitting deflection angle of the micro-LED, the theoretical value requirements of $\theta_{n}$ can be met. Eventually, the naked-eye 3D display can be realized.

### 2.2. Structural Design of Parallel Light-Deflecting Metasurface

**Figure 2:**
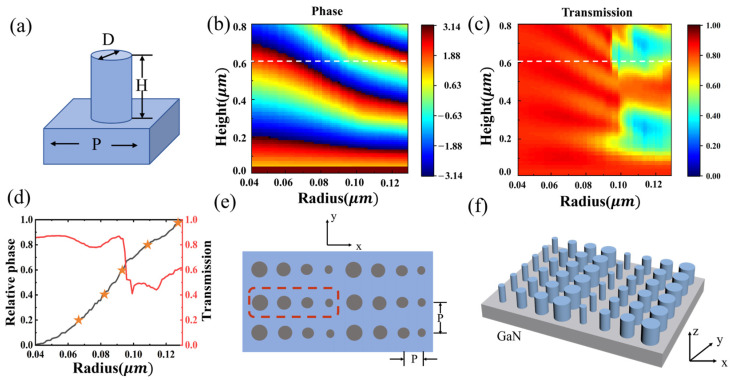
(**a**) Schematic diagram of the unit structure. (**b**) Simulated phase shift as a function of nanorod radius and height. (**c**) The obtained transmittance diagrams of the nanorod with the change in diameter and height. (**d**) Phase and transmittance corresponding to different radius unit structures. (**e**) Top view of the deflected metasurface (The red dashed box represents one cycle of the metasurface). (**f**) A 3D stereo schematic of the deflected metasurface.

Based on the principle of transmission-phase, we achieve phase modulation by varying the geometrical parameters of the medium structure within the period of the metasurface cell, and then changing the equivalent refractive index in the range. The light source is set to a 532 nm plane wave, at which GaN has a high refractive index of 2.34 and a significant energy bandgap of 3.4 eV, which effectively avoids interbond jumps in the visible spectrum. Thus, the parallel light deflection metasurface is formed by a series of GaN cylindrical nanorod units arranged in periodic repetitions. The symmetric structure ensures that it is polarization-independent, as shown in Figure 2a. The period of the nanorods is set to 300 nm, which is about half of the wavelength, to satisfy the equivalent medium requirement and the weak electromagnetic coupling between the nanorods. We used the S-parameter extraction method in the finite-difference time-domain (FDTD) simulation from Lumerical to build a structural phase library to relate the phase shift and transmittance of the cell with its geometrical parameters, including height, radius, and period. We obtained an image of its phase shift and transmittance versus the nanorod’s height and radius, as shown in Figure 2b,c. It can be found that when the height of the nanorod is taken as 600 nm, the phase shift variation over the entire range of 0–2π can be achieved when the radius is varied in the range of 40–130 nm and the transmittance is high.

The construction of an oblique wavefront is required to enable the metasurface to directionally deflect the beam after the incident of parallel light. The relative phase shift between neighboring nanorods in one cycle is to be satisfied:

(2) $$∆\varphi =\frac{2\pi }{\lambda }sin\theta \cdot p$$

where the ∆φ is the relative phase shift between the nanorods, λ is the working wavelength of the incident light, θ is the angle of directional deflection of the beam, and p is the period of the individual nanorods. Relative phase shifts are introduced to classify the phase shift variations of the super unit into discrete values such as 0.4π, 0.8π, 1.2π, 1.6π, and 2π over the entire range of 2π. Then, by selecting nanorods with different radii to satisfy the phase shift dispersion value at a specific position, beam deflection at an arbitrary angle can be achieved. For example, to achieve a beam deflection of about 20 degrees at 532 nm, five nanorods with different radii are required to form a cycle, as shown by the orange pentagram markers in Figure 2d. Figure 2e and Figure 2f, respectively, show a top view and a 3D schematic of a deflected metasurface structure. The red dashed line in Figure 2e represents one cycle of the metasurface.

### 2.3. Design of Deflected Metasurface for Point Light Sources

**Figure 3:**
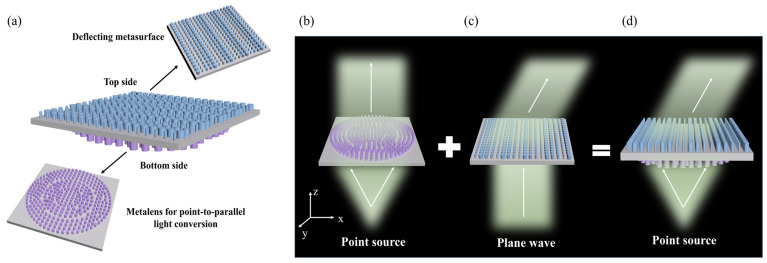
(**a**) Schematic diagram of the double-layer metasurface structure. (**b**) Point light source collimating metalens. (**c**) Parallel light deflection metasurface. (**d**) Point light source deflected double-layer metasurface.

We know that the traditional micro-LED light source has a wide Lambertian distribution pattern with no definite luminous direction. Therefore, we propose a double-layer metasurface structure design method, as shown in Figure 3a. The lower surface is a collimating metalens, which can convert the micro-LED incident light from a point source into parallel light, and the upper surface is a deflecting metasurface, which can deflect the incident light according to the required angle. The combination of the double-layer structure can realize the parallel alignment and deflection of the micro-LED light source. In this way, when two or more deflection angles can be realized, parallax can be formed, and thus depth perception can be formed when entering the human eyes.

In contrast to conventional lenses that rely on gradual phase accumulation in a refractive medium to focus light, metalens can achieve focusing by abrupt phase modulation across subwavelength elemental units. The input plane wave is modulated into a convergent wavefront through the metalens, and the required phase of each nanopillar at the (x, y) position must satisfy the following equation:

(3) $$\Phi \left(x,y\right)=\frac{2\pi }{\lambda }\left(f-\sqrt{f^{2}+x^{2}+y^{2}}\right)$$

where λ is the design wavelength (λ = 532 nm), f is the target focal length (f = 12.12 µm), and x and y are the positional coordinates of each nanopillar relative to an origin at the center of each lens. The nanopillar diameter can be adjusted to achieve the desired phase profile. In this method, phase accumulation is achieved by the waveguide effect; the height of the nanopillar should be high enough to provide a phase coverage of 2π over a certain range of diameter variations.

Then, using the principle of reversibility of the optical path, when a diverging point light source is placed at the focal point of the focusing metalens, the metalens can convert the diverging light into a collimated beam, realizing the conversion of the point light source to a parallel light source, as shown in Figure 3b. Then, the outgoing parallel light is incident on the deflecting metasurface designed according to the principle in Section 2.2, as shown in Figure 3c. The dual-layer metasurface superposition can effectively realize the directional deflection of the micro-LED light source, as shown in Figure 3d. It is worth noting that the bilayer structure seems to have a common substrate, but it is a stack of two-unit structure substrates, while the unit structure used to construct the phase library is a combination of the substrate and the nanopillar. Therefore, the constructed phase libraries are the result of light modulation by the nanopillars of different radii and the substrate.

## 3. Results and Discussion

### 3.1. Naked-Eye 3D Display System under Parallel Light Incidence

Since the main applications of micro-LED displays are computers, mobile phones, etc., the distance from the human eye to the display is set to 250 mm, and the distance between the human eyes is calculated as 65 mm. The system contains two deflected metasurface array structures, and each subpixel is set to be 0.084 mm × 0.084 mm. Based on the analysis of the previous structural design and methodology, the specific parameters of the naked-eye 3D display system are determined as shown in Table 1.

For the subpixel that puts the light into the left eye, the distance between the two adjacent left-eye viewpoints should be 130 mm. Based on this, we performed a detailed calculation based on Equation (1) for both eyes’ subpixels, and the results are shown in Table 2. In the simulation, the light source was set as X-polarized parallel light at 532 nm. Perfectly matched layer (PML) boundary conditions were used in the X, Y, and Z directions to eliminate boundary effects. An X-Y plane monitor is placed directly above the metasurface to collect the scattered light directly above it, and the far-field results are obtained using the far-field command. A virtual receiver screen is placed at the optimal observation distance to replace the human eye.

**Figure 4:**
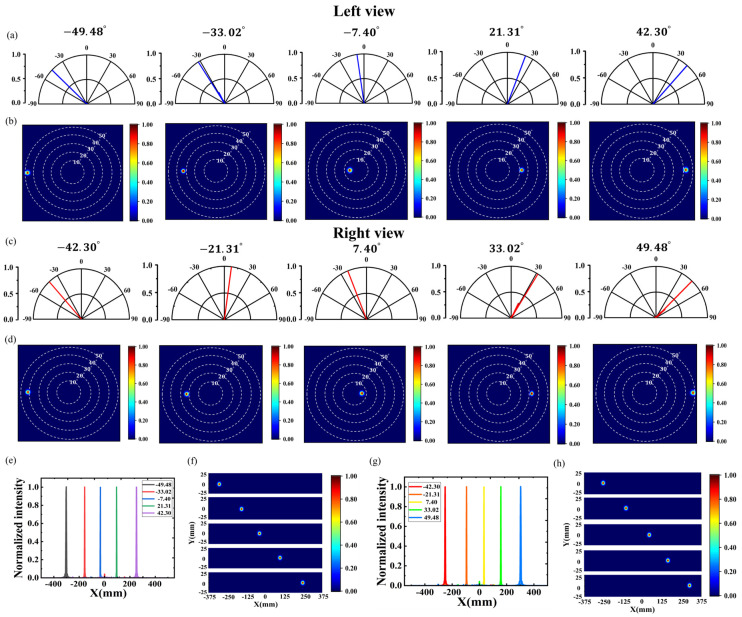
Single-angle parallel light deflection metasurfaces. (**a**–**d**) Far-field polar plots and far-field patterns of left-eye and right-eye subpixel deflections, respectively. (**e**–**h**) Normalized light intensity distribution and far-field in the X-Y plane of left-eye and right-eye subpixel deflections, respectively.

According to the calculation results in Table 2 and the working principle of the parallel light deflecting metasurface in Section 2.2, we used the periodic arrangement of nanopillar units with different radii to form the deflecting metasurface. When the deflection angle increases, the period of the nanopillars is reduced to increase the number of nanopillars, effectively avoiding the decrease in deflection efficiency. Firstly, the outgoing emissions from a single angle of the left-eye and right-eye subpixels were simulated. The five figures in Figure 4a show the far-field polar plots of the left-eye subpixel metasurface deflected by ${-49.48}^{{}^{\circ}}$, ${-33.02}^{{}^{\circ}}$, ${-7.40}^{{}^{\circ}}$, $21.31^{{}^{\circ}}$, and $42.30^{{}^{\circ}}$ from the top to the bottom, respectively, and the far-field pattern maps of the five angles are shown in Figure 4b. The far-field conditions of the right-eye subpixel deflections of ${-42.30}^{{}^{\circ}}$, $-21.31^{{}^{\circ}}$, $7.40^{{}^{\circ}}$, $33.02^{{}^{\circ}}$, and $49.48^{{}^{\circ}}$ are shown in Figure 4c,d. The results show that the designed metasurface can be accurately oriented and deflected as needed.

Figure 4e,g shows the effect of different deflection angles on the normalized radiant light intensity distribution for the left-eye subpixel and the right-eye subpixel when y is 0, respectively. Every time the beam deflection angle is changed from angle n to angle n + 1, the position of the maximum radiation intensity is shifted to the right by 130 mm, which is exactly twice the distance between the human eyes. Figure 4f,h shows the far-field radiated light intensity distributions in the X-Y plane for the left-eye subpixel and the right-eye subpixel at the optimal observation distance, respectively; the actual beam deflection positions are not far from the expected theoretical values. The results show that when the beam deflection angle of a pixel is changed, the position of beam transmission can be changed correspondingly with less stray light, and high fidelity of the image can be achieved.

The deflection efficiency is defined as the fraction of optical power collected within the angle of $\theta \pm 5^{{}^{\circ}}$ with respect to the total transmitted optical power, where θ is the deflection angle. For example, for the deflected metasurface with left-eye subpixel deflection angles of $-7.40^{{}^{\circ}},21.31^{{}^{\circ}}$, the collected optical power was calculated at ${-12.40}^{{}^{\circ}}$∼${-2.40}^{{}^{\circ}}$,$\;16.31^{{}^{\circ}}$∼$26.31^{{}^{\circ}}$ respectively. For the deflected metasurface with right-eye subpixel deflection angles of ${-42.30}^{{}^{\circ}},33.02^{{}^{\circ}}$, the optical power collected at $-47.30^{{}^{\circ}}$∼${-37.30}^{{}^{\circ}}$, $27.02^{{}^{\circ}}$∼$38.02^{{}^{\circ}}$ respectively, was calculated. The total transmitted optical power is defined as all the optical power collected within the range of ${-90}^{{}^{\circ}}∼90^{{}^{\circ}}$. Similarly, the deflection efficiencies were calculated for each of the ten angles, as shown in Table 2. The ultra-high deflection efficiencies indicate that it is possible to achieve efficient and precise deflection at any angle within the range of $-50^{{}^{\circ}}$∼$50^{{}^{\circ}}$. In addition, for the left-eye subpixel deflection of n degrees and the right-eye subpixel deflection of -n degrees, the phase shift required for the wavefront is equal in magnitude and opposite in direction, so that the nanorod radius values are the same and the arrangement directions are opposite within one cycle. Therefore, the deflection efficiency of the two angles is approximately equal. For example, the efficiency of left-eye subpixel deflection by $-7.40^{{}^{\circ}}$ is calculated to be 90.78%, and the efficiency of right-eye subpixel deflection by $7.40^{{}^{\circ}}$ is 91.05%.

**Figure 5:**
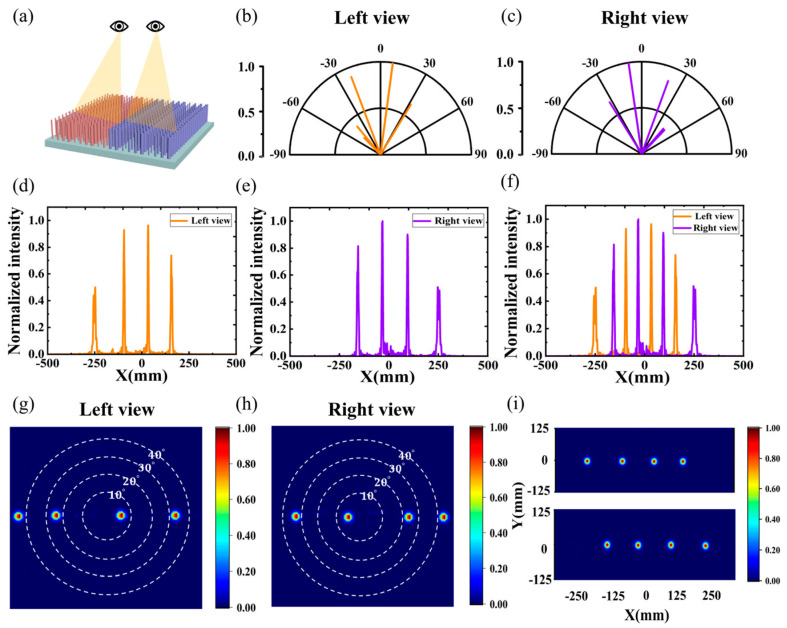
(**a**) Schematic diagram of dual-view naked-eye 3D display. (**b**,**c**) Far-field polar plots of the left and right viewing areas. (**d**,**e**) Normalized light intensity distribution of the left and right viewing areas. (**f**) Binocular normalized radiation light intensity distribution. (**g**,**h**) Far-field patterns of the left and right viewing areas. (**i**) Far-field radiance maps of the left and right viewing areas in the X-Y plane.

To analyze the naked-eye 3D display effect for both eyes, it is necessary to construct and simulate the left- and right-eye auto-stereoscopic 3D parallel light models, respectively, as shown in Figure 5a, where the red and violet nanopillars represent the subpixels of the light beams deflected towards the left and right eyes, respectively. When simulating the left-eye pixels, the left-eye pixels can be fully imaged, and the right-eye pixels are all turned off, same for the right eye. In real life, the application range of a two-viewpoint 3D display system is relatively narrow, and cannot meet the demand of multiple people watching at the same time. Therefore, it is necessary to research and develop naked-eye 3D display technology with multiple viewpoints and large fields of view.

By dividing a subpixel into four modules, the nanopillar of each module realizes four different angles, respectively, thus realizing the simultaneous outgoing of parallel beams with multiple angles. Figure 5b,g shows the far-field polar plots and far-field pattern plots of a left-eye subpixel emitting ${-42.30}^{{}^{\circ}}$, ${-21.31}^{{}^{\circ}}$, $7.40^{{}^{\circ}}$, and $33.02^{{}^{\circ}}$ at the same time, respectively, and the beams were able to deflect the preset angle effectively. The calculated beam deflection efficiency for each angle is shown in Table 3, and the total deflection efficiency of one chip can reach 88.40%. The multiple simultaneous outgoing angles of the right-eye subpixels are shown in Figure 5c,h, with a total deflection efficiency of 88.52%.

Figure 5d,e shows the normalized radiant light intensity distributions of the left-eye view and right-eye view, respectively. The merged binocular view is shown in Figure 5f, where the peaks of the adjacent left-eye view are 65 mm apart from the peak of the right-eye view, which is in line with the spacing of the human eye. This indicates that the left and right viewpoints can be better separated without additional optical elements. It can be found that the distribution of radiated light intensity in the multi-viewpoint viewing area shows a crest and trough pattern, with the peak at the viewing position, which has the highest intensity, and there is no crossover between the neighboring peaks, which can achieve zero crosstalk. Figure 5i shows the far-field radiated light intensity distributions in the X-Y plane for the left-eye subpixel and the right-eye subpixel at the optimal viewing distance. As a result, a binocular naked-eye 3D system with a single light source, a large field of view, multiple viewpoints, high resolution, and high optical efficiency under parallel light was realized.

### 3.2. Realization of the Collimated Metalens

**Figure 6:**
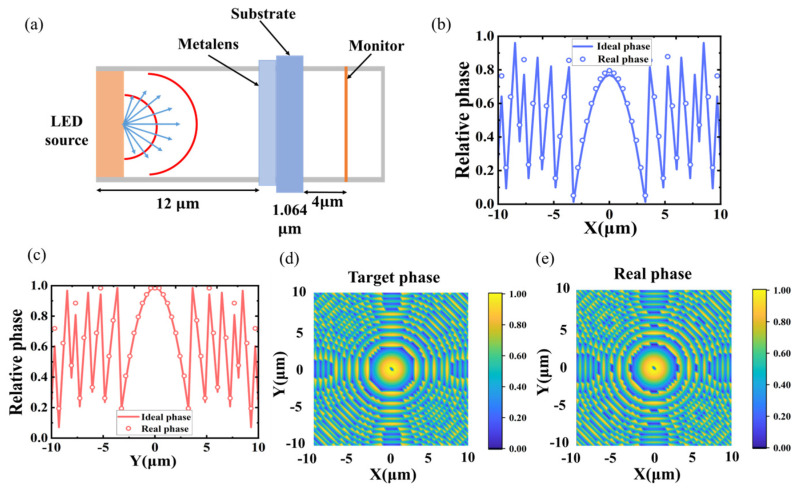
(**a**) Structural diagram of the collimated metalens model. (**b**,**c**) Matching diagram of the actual phase and ideal phase in the X-direction at y = 0 and in the Y-direction at x = 0, respectively. (**d**,**e**) Ideal and actual phase distribution in the X-Y plane, respectively.

A design principle similar to the parallel light deflection metasurface unit structure is adopted, using GaN cylindrical nanorods as the basic unit structure. The height of the unit structure was set to 600 nm, the period was set to 400 nm, and the radius of the nanorods was varied in the range of 40 nm–130 nm. The target phase at each position coordinate was then calculated using MATLAB R2019b software, the actual phase was matched with the desired phase, and the corresponding structural dimensions were obtained by phase indexing in the structure phase library to obtain the corresponding structural dimensional pickup values. Figure 6b,c shows the matching of the actual and ideal phases in the x- and y-directions at y = 0 and x = 0, respectively, where the solid line represents the ideal phase, and the hollow circles indicate the actual phase that can be provided by the nanopillar selected at each positional coordinate. The high degree of matching between the actual phase and the ideal phase indicates the reasonableness and feasibility of the nanopillar structure selection. Figure 6d,e shows the ideal phase distribution and the actual phase distribution of the lens in the X-Y plane design, respectively, showing similar symmetric concentric circle profiles.

Subsequently, the full-field model can be constructed in FDTD. The complete structure is shown in Figure 6a, with the *Z*-axis positive direction above the structure, the metalens is placed below the substrate with the diameter size set to 42 µm and the focal length set to 12.12 µm. Since micro-LED light sources are usually described as many point sources with Lambertian intensity profiles, this is not a coherent result. Therefore, in this study, a square GaN substrate of 42 μm size and a dipole light source with a wavelength of 532 nm was used instead of a micro-LED light. Monitors were placed above the light source and the metalens, and the boundary conditions were set to a metallic boundary to minimize unnecessary losses.

**Figure 7:**
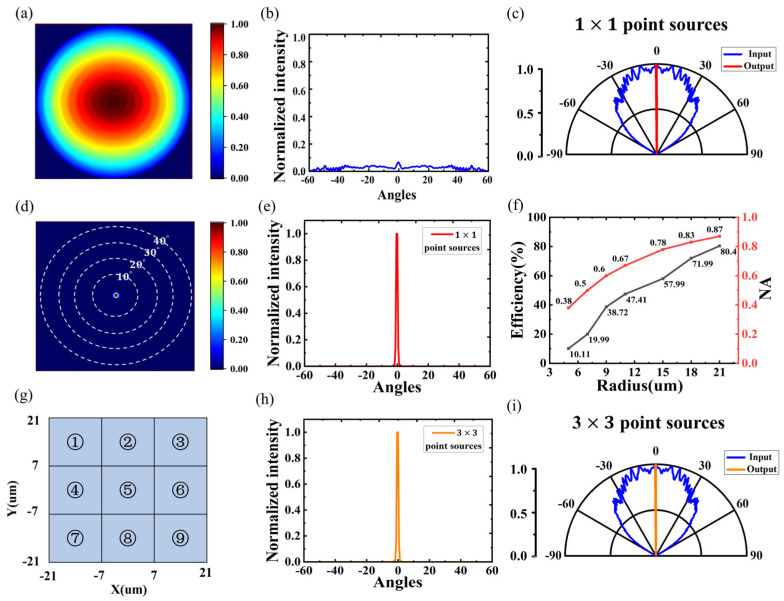
(**a**,**b**) Far-field patterns and normalized intensity distribution of a bare LED without any metalens. (**c**) The far-field polar plots of a 1 × 1 point light source array after being collimated by metalens. (**d**,**e**) Far-field patterns and normalized intensity distribution of micro-LED with integrated collimated metalens. (**f**) Variation of output luminous efficiency and numerical aperture with radius size. (**g**) A 3 × 3-point light source array structure. (**h**,**i**) Normalized intensity distribution and far-field polar plots of the outgoing light from a 3 × 3-point source array collimated by a metalens.

The outgoing light from the dipole source monitored by the monitor is shown in Figure 7a,b, and the intensity distribution of the transmitted light is similar to that of the Lambertian intensity distribution, so we can describe it in terms of a point source. The evanescent spherical wave emitted from the point source is collimated into parallel light by the 1 × 1 point source after passing through the designed metalens, as shown in Figure 7c, and the outgoing light is concentrated near the 0-degree angle. Figure 7d,e demonstrates the far-field pattern map and normalized radiation intensity map of the outgoing light, and it is calculated that 94.57% of the outgoing light is distributed within the range of $-1.2^{{}^{\circ}}\sim 1.2^{{}^{\circ}}$. Meanwhile, the effect of the metalens size on the efficiency of the outgoing light was explored. The size of the light source is set to 20 μm and kept constant, and the radius of the metalens is gradually increased from 5 μm to 21 μm. It can be seen from Figure 7f that the efficiency of the outgoing light of the metalens is gradually increased with the increase of the lens radius, and the numerical aperture is also increased. The results show that as the size of the metalens above the light source increases, more light outgoing from the light source will pass through the metalens before being transmitted to the monitor above.

However, a micro-LED light source is usually an incoherent, unpolarized surface light source that is composed of multiple point sources. Therefore, we designed a 3 × 3 array of point light sources, each placed at the center of the lattice, as shown in Figure 7g. Individual simulations are first performed for each point source, and then the results of each simulation are summed non-coherently to obtain the total result for a 3 × 3 array of point sources. Figure 7h,i shows the parallel source outgoing results for a 3 × 3 dipole light source array collimated by a metalens, exhibiting similar light emission to a single point source of the same size in Figure 7e, with a peak maximum of 1.522 × 10^−12^, and a single point source with a peak maximum of 1.520 × 10^−12^, which exhibits only an increase in intensity. The results indicate that the effect of the relative position of the light sources is negligible in a 42 μm light chip, thus it is feasible to use a single point source instead of a micro-LED light source for this study.

### 3.3. Naked-Eye 3D Display System under the Incidence of Point Light Source

After setting up the deflection metasurface and collimated metalens, the deflection metasurface is placed on top of the substrate to form a double-layer metasurface structure. The size of each subpixel is set as 0.042 mm × 0.042 mm. Other display parameters are set the same as those of the parallel light deflection 3D display system, and the pixel density PPI of this system can be calculated to be as high as 605.

**Figure 8:**
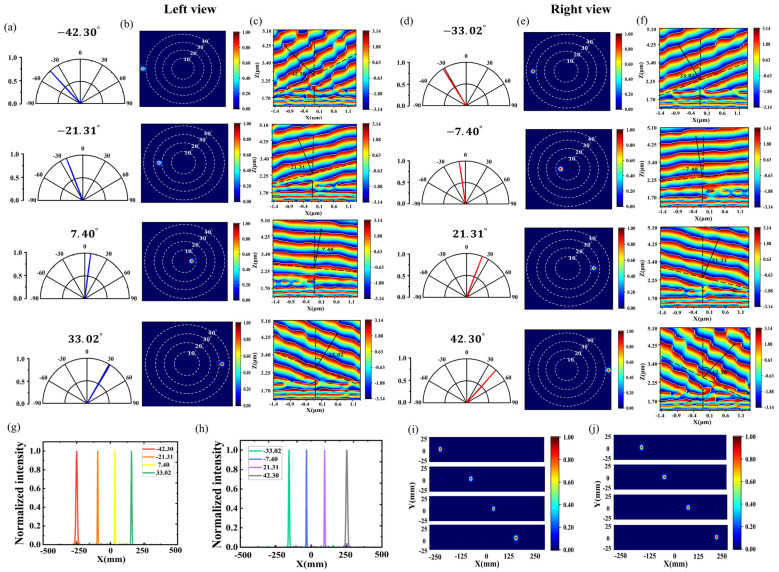
Single-angle point light source deflection metasurfaces. (**a**,**b**) Far-field polar plots and far-field patterns of left-eye subpixel deflections, respectively. (**c**,**f**) Phase profiles for specific emission angles of left-eye subpixels and right-eye subpixels, respectively. (**d**,**e**) Far-field polar plots and far-field patterns of right eye-subpixel deflections, respectively. (**g**,**i**) Normalized light intensity distribution and far-field in the X-Y plane of left-eye subpixel deflections, respectively. (**h**,**j**) Normalized light intensity distribution and far field in the X-Y plane of right-eye subpixel deflections, respectively.

Firstly, the single-angle emission of the left-eye subpixel and the right-eye subpixel were simulated, respectively, and the setting and calculation of the beam deflection angle and the theoretical deflection position were the same as those in the previous section. The eight figures in Figure 8a,b show, from top to bottom, the far-field polar plots and radiation pattern plots of the left-eye subpixels deflected by ${-42.30}^{{}^{\circ}}$, ${-21.31}^{{}^{\circ}}$, $7.40^{{}^{\circ}}$, and $33.02^{{}^{\circ}}$, respectively, under the illumination of the point light source, which can be seen to be able to accurately orientate the deflections as required after passing through the double-layered metasurface. The point light source is also limited to a narrow angular range by the initial wide Lambertian angular distribution. Figure 8c shows the phase profile of the metasurface obtained from the X-Z surface monitor, which clearly shows that the point light source is first converted to a plane wave by the collimating lens on the lower surface, with the wavefront almost parallel to the transverse cross-section, and then the beam passes through the deflected metasurface on the upper layer, where the wavefront is transformed into a series of plane waves with a specific tilt angle.

Correspondingly, Figure 8d–f shows the far-field polar plots, radiation pattern plots, and X-Z plane phase profiles of the right-eye subpixels deflected by ${-33.02}^{{}^{\circ}}$, ${-7.40}^{{}^{\circ}}$, $21.31^{{}^{\circ}}$, and $42.30^{{}^{\circ}}$, respectively, under the illumination of a point source, which are calculated to have high deflection efficiencies of large versus 80% for each angle, as shown in Table 4. Figure 8g,h shows the effect of different deflection angles on the normalized radiation intensity distributions for the left- and right-eye subpixels when y is 0. The far-field radiated light intensity distributions of the left-eye subpixel and the right-eye subpixel in the X-Y plane at the optimal observation distance were also obtained using the monitor, as shown in Figure 8i,j. The actual beam deflection position is not far from the expected theoretical value, indicating that the point light source achieves a specific positional transmission by the directional angle of deflection.

**Figure 9:**
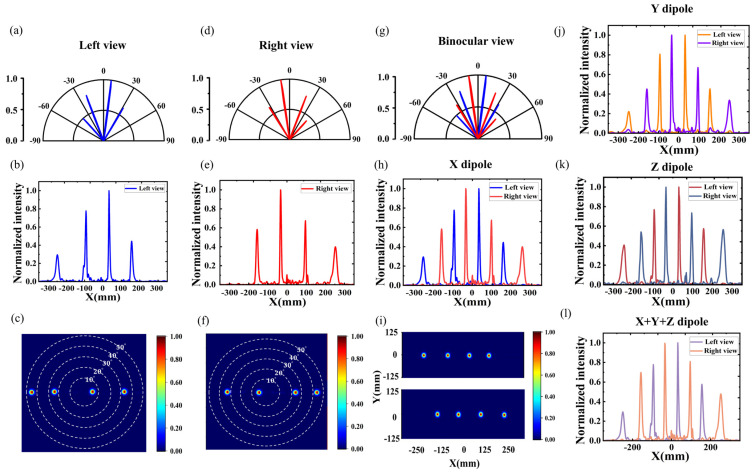
(**a**,**d**) Far-field polar plots of the left and right viewing areas. (**b**,**e**) Normalized light intensity distribution of the left and right viewing areas. (**c**,**f**) Far-field patterns of the left and right viewing areas. (**g**–**i**) Far-field polar plots, normalized light intensity distribution, and X-Y plane far-field radiation plots for binocular views. (**j**,**k**) Normalized light intensity distribution in the y- and z-polarized directions. (**l**) Normalized light intensity distribution when the light source is an electric dipole with three orthogonal polarization directions.

Based on the good implementation of a single angle, the left- and right-eye auto-stereoscopic 3D point light source deflection models with multiple viewing angles were constructed and simulated separately. By dividing a subpixel into multiple modules, under the illumination of a point light source, a left-eye subpixel simultaneously emits ${-42.30}^{{}^{\circ}}$, ${-21.31}^{{}^{\circ}}$, $7.40^{{}^{\circ}}$, and $33.02^{{}^{\circ}}$, and a right-eye subpixel simultaneously emits $-33.02^{{}^{\circ}}$, ${-7.40}^{{}^{\circ}}$, $21.31^{{}^{\circ}}$, and $42.30^{{}^{\circ}}$, as shown in Figure 9a–f. The beam was accurately deflected at the preset position. The calculated beam deflection efficiencies for each angle are shown in Table 5, and the total deflection efficiencies of the two chips are as high as 85.74% and 80.46%, respectively. The binocular view can be obtained by combining the left-eye view and the right-eye view, as shown in Figure 9g–i. At the same time, the point light source was changed from the X-polarization direction to the Y-polarization direction and Z-polarization direction, respectively, to explore whether the polarization of the light source had any effect on the results, as shown in Figure 9j,k. The results under the irradiation of three orthogonal polarization direction electric dipole light sources are summed up and shown in Figure 9l. The results show that the bilayer metasurface structure exhibits similar outgoing results regardless of the polarized light source irradiation, proving its polarization insensitivity.

At the same time, the narrower emission peaks indicate that the micro-LED light source with a large divergence angle has a reduced light-emitting half-angle after passing through the double-layer metasurfaces, which consequently reduces the beam width on the receiving screen and reduces stray light. This suggests that a smaller light-emitting half-angle results in more concentrated light with lower loss and higher resolution. This solves the problem of large crosstalk caused by beam interference between pixels when conventional micro-LEDs are used as display pixels due to their large divergence angle. At the same time, it achieves a large angle from −45 degrees to 45 degrees and high-efficiency emission, thus designing a naked-eye 3D display system with a simple structure, large field of view, multiple viewpoints, and high resolution. However, it can also be observed that there will be a gap between the emission peaks of only two subpixels, thus creating a visual viewing blind spot, so the visual blind spot can be eliminated by adding more subpixels and, based on the same design methodology, deflecting the light emitted from these subpixels to the position where there is a gap.

## 4. Conclusions

In summary, we propose a micro-LED naked-eye 3D display system based on metasurface. By introducing a double-layer metasurface thin film structure, the collimated metalens in the lower layer converts the micro-LED point light source from a wide Lambertian distribution to parallel light outgoing. The parallel light deflection metasurface with modular design on the upper layer achieves 8 angles in the range of $-45^{{}^{\circ}}\sim 45^{{}^{\circ}}$ and high-efficiency directional emission greater than 80% and low crosstalk. Meanwhile, the pixel density of the monitor is as high as 605 PPI. The peak values of the adjacent left eye and right eye views are 65 mm apart, which conforms to the distance between human eyes and can effectively meet the imaging requirements of a naked-eye 3D display. By non-coherent summation of a 3 × 3 point light source array, the feasibility of using a single-point source instead of a micro-LED light source for research was verified. This innovative method simplifies the device structure, minimizes crosstalk, and ensures large-angle, multi-viewpoint, and high-efficiency 3D display imaging without sacrificing resolution. It demonstrates the great development potential of naked-eye 3D display applications.

## Figures and Tables

**Table 1:** Simulation parameters of the parallel light deflection 3D display system.

System Parameter	Numerical Value
Distance between eyes (D)	65 mm
Number of viewpoints (K)	8
Optimal observation distance (L)	250 mm
Pixel size	0.084 mm × 0.084 mm
Acceptance screen size	1000 mm × 1000 mm
Resolution (R)	1920 × 1080
Pixel density (PPI)	300

**Table 2:** Single-angle parallel light deflection metasurface angle efficiency calculation.

	Perspectives	1	2	3	4	5
Left-eye subpixel	Theoretical beam deflection position	−292.5 mm	−162.5 mm	−32.5 mm	97.5 mm	227.5 mm
	Beam deflection angle	${-49.48}^{{}^{\circ}}$	${-33.02}^{{}^{\circ}}$	${-7.40}^{{}^{\circ}}$	$21.31^{{}^{\circ}}$	$42.30^{{}^{\circ}}$
	Deflection efficiency	91.83%	89.42%	90.78%	89.00%	88.59%
Right-eye subpixel	Theoretical beam deflection position	−227.5 mm	−97.5 mm	32.5 mm	162.5 mm	292.5 mm
	Beam deflection angle	${-42.30}^{{}^{\circ}}$	$-21.31^{{}^{\circ}}$	$7.40^{{}^{\circ}}$	$33.02^{{}^{\circ}}$	$49.48^{{}^{\circ}}$
	Deflection efficiency	89.99%	89.00%	91.05%	89.42%	91.83%

**Table 3:** Deflection efficiency of multi-view parallel light deflecting metasurface.

	Perspectives	1	2	3	4	Total Efficiency
Left perspective	Beam deflection angle	${-42.30}^{{}^{\circ}}$	$-21.31^{{}^{\circ}}$	$7.40^{{}^{\circ}}$	$33.02^{{}^{\circ}}$	88.40%
	Deflection efficiency	17.72%	24.06%	23.61%	23.01%	
Right perspective	Beam deflection angle	${-33.02}^{{}^{\circ}}$	${-7.40}^{{}^{\circ}}$	$21.31^{{}^{\circ}}$	$42.30^{{}^{\circ}}$	88.52%
	Deflection efficiency	23.32%	25.15%	22.55%	17.50%	

**Table 4:** Calculation of deflection efficiency of deflected metasurface of single-angle double-layer point light source.

	Perspectives	1	2	3	4
Left-eye subpixel	Beam deflection angle	${-42.30}^{{}^{\circ}}$	$-21.31^{{}^{\circ}}$	$7.40^{{}^{\circ}}$	$33.02^{{}^{\circ}}$
	Deflection efficiency	83.63%	83.21%	92.25%	89.70%
Right-eye subpixel	Beam deflection angle	${-33.02}^{{}^{\circ}}$	${-7.40}^{{}^{\circ}}$	$21.31^{{}^{\circ}}$	$42.30^{{}^{\circ}}$
	Deflection efficiency	89.67%	92.26%	88.02%	83.59%

**Table 5:** Calculation of efficiency of deflected metasurfaces for multi-viewpoint light sources.

	Perspectives	1	2	3	4	Total Efficiency
Left perspective	Beam deflection angle	${-42.30}^{{}^{\circ}}$	$-21.31^{{}^{\circ}}$	$7.40^{{}^{\circ}}$	$33.02^{{}^{\circ}}$	85.74%
	Deflection efficiency	13.97%	27.92%	25.03%	18.82%	
Right perspective	Beam deflection angle	${-33.02}^{{}^{\circ}}$	${-7.40}^{{}^{\circ}}$	$21.31^{{}^{\circ}}$	$42.30^{{}^{\circ}}$	80.46%
	Deflection efficiency	20.83%	23.88%	17.95%	17.80%	

## Data Availability

Data are contained within the article.
